# Anti-DLL4, a cancer therapeutic with multiple mechanisms of action

**DOI:** 10.1186/2045-824X-3-18

**Published:** 2011-08-10

**Authors:** Austin Gurney, Timothy Hoey

**Affiliations:** 1 OncoMed Pharmaceuticals, 800 Chesapeake Drive, Redwood City, California 94063, USA

## Abstract

DLL4 is a ligand for the Notch family of receptors. DLL4 has many important functions in normal development and tissue homeostasis, including roles in the immune system, the gastro-intestinal tract, and in vascular development. Because of the importance of Notch signaling in stem cell biology, DLL4 has been investigated for its role in the maintenance and proliferation of cancer stem cells (CSC). In addition, its important role in angiogenesis has been investigated for utility as an anti-angiogenic agent. Preclinical studies have highlighted that both anti-CSC and anti-angiogenic activities contribute to its anti-tumor efficacy, and have supported the clinical development of anti-DLL4 antibody for the treatment of cancer.

## Introduction

Cancer can be considered as an aberrant and mutant recapitulation of normal tissue development and homeostasis. Tumors are typically highly heterogeneous at the cellular level, and this heterogeneity frequently mirrors the cellular heterogeneity of the normal tissue. Normal tissue development and homeostasis is driven by an organized hierarchy of stem and progenitor populations which give rise to various differentiated cell types with specialized functions. Long term tissue maintenance is enabled by the unique ability of the stem cell to exhibit self-renewal, which is defined as the ability to proliferate while maintaining pluripotency. Similarly, cancer cells inappropriately activate self-renewal pathways and this enables their ability to grow indefinitely. Thus, the potential connections between normal stem cells and cancer have great importance for understanding tumor biology and also for developing new therapeutic strategies. In the past decade it has become increasingly clear that this self-renewal property is not possessed by all cells within a tumor, but that there exists a subpopulation of cells, often referred to as "cancer stem cells" or "tumor initiating cells" which possesses the ability to undergo self-renewal and thereby drive the growth of the tumor [1-3]. These cells also possess the ability to initiate the growth of new tumors that recapitulate the heterogeneity of the parent tumor. Thus, these cells have hallmark capabilities analogous to normal stem cells. This relationship has been strengthened by genetic studies which have shown that normal stem cells can be the cell of origin for tumors [4]. Additionally, cancer initiating mutations can originate in more differentiated cells and confer stem-like properties on the tumor cells. These connections have led to a careful examination of stem cell signaling pathways and their role in cancer. Intriguingly, several of these pathways, including the Notch and Wnt pathways, have long been recognized to be activated by oncogenic mutations and disregulated in cancer.

In addition to cancer's need to achieve the stem cell-like property of self-renewal, a cancer must also recruit a support system of stroma and vasculature. The development of vasculature is a complex developmental process, analogous to the development of organs, and so it is not surprising to note that here too signaling pathways important to stem cells, including Notch, have important role in cell fate decisions. There are roles recognized for multiple Notch receptors and multiple Notch ligands within this process [5-8]. These molecules play roles both within the endothelial cell layer, where they are involved in vessel branching and maturation, and in the surrounding pericyte and smooth muscle layers. Jagged1 and Notch3 are of particular importance in pericyte function [9-11]. DLL4 acting through Notch1 and Notch4 appears to play key roles regulating endothelial cells and bone marrow-derived endothelial cell progenitors during normal and tumor angiogenesis [12,13].

These two lines of research, the role of the Notch pathway in the maintenance of cancer stem cells, and the activity of Notch in tumor vasculature, have led to intense research interest in targeting components of this pathway for the development of novel therapeutics. Through this effort, DLL4 has emerged as a compelling target. Indeed, an antibody to DLL4, OMP-21M18, was the first therapeutic entity that selectively targeted the Notch pathway to enter human clinical trials. Gamma-secretase inhibitors (GSIs) that inhibit the ligand-dependent cleavage of Notch receptors have also been developed as anti-cancer therapies. Treatment with GSIs has been found to result in severe gastrointestinal toxicity, limiting their therapeutic utility, due to the combined inhibition of both Notch1 and Notch2 within the stem-progenitor compartment of the intestinal crypt [14,15]. In addition to processing Notch proteins, gamma-secretase cleaves many other membrane proteins and is involved in a large number of signaling pathways apart from Notch, and these pleiotropic effects are also likely to contribute to the toxicity of GSIs [16,17].

DLL4 is one of three delta-like ligands in the mammalian genome [18]. Similar to Drosophila Delta, it acts as an agonist ligand to modulate the activity of Notch receptors. Binding studies and in vitro signal transduction studies indicate that DLL4 is readily able to signal through each of the four human Notch receptors, demonstrating its potential to participate in many of the diverse functions that have been described for the Notch family. Elucidating the full range of its activities is an ongoing area of investigation, and is somewhat complicated by the potential for compensatory action by other Notch ligands. Further complicating this analysis is the potential for DLL4 to be expressed in minor subsets of cells, such as stem cells and progenitors, which may lead to an under-appreciation of DLL4 expression and its relevance to a particular tissue. LacZ reporter studies in developing mouse embryos have revealed expression with the vascular system, multiple structures within the nervous system, the gastrointestinal system, the glomeruli of the kidney, and the thymus [19] Gene disruption studies indicate that DLL4 plays an important role in angiogenesis - haploinsufficiency results in embryonic lethality due to angiogenic defects [20-22]. Conditional gene disruption studies have also demonstrated critical roles for DLL4 in T cell development [23,24]. In addition, DLL4 is also expressed by myeloid cells including macrophage and dendritic cells and plays a role in modulating adaptive immune response [25-27]. DLL4 is one of the ligands important for lineage commitment within the epithelial cell lining of the gastrointestinal tract. Interestingly, DLL1 and DLL4 appear to have redundant functions in normal intestinal development and homeostasis [28], which accounts for the fact that selective inhibition of DLL4 has minimal impact on the function of the GI tract.

### Mechanism of DLL4 in Angiogenesis

Several groups have reported the discovery of antibodies which block the ability of DLL4 to activate Notch [29-31]. This work contributed to the discovery that DLL4-Notch signaling is part of a negative feedback loop in the angiogenic process. Blockade of DLL4-Notch binding or loss of function of DLL4 leads to an up-regulation of VEGF signaling resulting in deregulated, hyperproliferation of the tip cells ultimately leading to immature vessels that lack a functional lumen. It has been shown that DLL4 inhibition can have a widespread effect in reducing tumor growth in a number of different xenograft models through this angiogenic mechanism [29,30]. Inhibition of VEGF signaling is now a well established strategy for anti-cancer therapeutics [32,33]. The work on DLL4 shows that up-regulation of VEGF in tumor vasculature can also have a therapeutic benefit. Tumors responsive to anti-DLL4 include those that are resistant to inhibition with anti-VEGF highlighting the potential utility of this approach.

We now have a fairly detailed understanding of the mechanism of action for how disrupting DLL4-Notch signaling leads to non-functional vasculature and thereby reduces tumor growth. Normally, DLL4-Notch signaling restricts the numbers of tip cells in response to VEGF, whereas inhibition of DLL4 leads to increased tip cell formation and reduced numbers of stalk cells in angiogenic regions [34]. Inhibition of DLL4 up-regulates VEGF expression as well as its receptors VEGFR2 and VEGFR3. The effect of DLL4 on sprouting angiogenesis is also mediated through regulation of matrix metalloproteinase (MMP) expression [35].

### Role of DLL4 in Tumor Initiating Cells

In addition to its role in the tumor vasculature, evidence has accumulated indicating an important role for DLL4-Notch signaling in tumor cells [36,37]. Across a range of major tumor types, including colorectal, kidney, breast and lung cancer, DLL4 protein expressed by tumor cells can be detected in a high percentage of patient samples [38]. An anti-DLL4 antibody, OMP-21M18, was found to have anti-tumor activity in patient-derived xenograft models independent of any effect on angiogenesis [27]. This antibody does not cross-react with murine DLL4 and therefore has no effect on the vasculature in murine xenografts. The combination of blocking DLL4 in tumor and vascular cells was shown to have an additive effect. Importantly, inhibition of DLL4 not only reduced tumor growth but also reduced tumor initiating cells as shown by serial transplantation experiments [31]. Treatment of colon tumors with anti-DLL4 up-regulates markers of more differentiated colon cells (for example, ATOH1 and Chromogranin A) indicating that DLL4-Notch inhibition limits the stem/progenitor-like properties of colon tumor cells and promotes a more differentiated phenotype. Potential insight into how DLL4-Notch signaling might function in colon cancer is provided by the fact that in normal colon development DLL4 is expressed on the paneth cells [39] which are adjacent to the stem cells and thought to play an important role as the niche to support stem cell maintenance and self-renewal through activation of Notch signaling.

Anti-DLL4 treatment was shown to have synergistic activity with various chemotherapeutic agents in reducing tumor volume and tumor initiating cell frequency [31]. These data are consistent with the hypothesis that the less differentiated, stem-like cancer cells are resistant to conventional cancer treatments including chemotherapy whereas promoting differentiation sensitizes tumor cells to the cytotoxic effects of chemotherapeutic drugs. More recently, anti-DLL4 was found to be active in a number of colon tumor xenografts including those harboring KRAS mutations which are insensitive to anti-EGFR treatment [40]. Since tumorigenic CSCs are thought to mediate tumor recurrence after treatment and the metastatic spread of the disease, agents that block key CSC self-renewal hold tremendous promise as improved cancer treatments.

### Perspective

Anti-DLL4 attacks cancers through two distinct mechanisms, an anti-angiogenic effect and a reduction of CSCs, each fundamental for malignant tumor growth. This target offers tremendous potential for improved efficacy in cancer treatment. As discussed above, inhibition of DLL4 signaling has been shown to result endothelial cell hyperproliferation and increased vascular sprouting in tumor angiogenesis. In rodents, DLL4 inhibition or loss of function has been associated with certain adverse events including vascular hyperproliferation in the liver and non-malignant vascular neoplasms [41,42]. These data raise concerns about potential toxicities associated with anti-DLL4 therapy in the clinic linked to its mechanisms of action in the vasculature and/or in stem cell biology. At least two anti-DLL4 antibodies have entered clinical testing (OMP-21M18 from OncoMed and REGN421 from Regeneron). To date there have not been reported incidents of either liver toxicity or vascular neoplasms in these clinical programs. None-the-less, DLL4 is an important ligand within a fundamental signaling pathway, and a molecule that robustly impacts vascular development, so it would not be surprising if adverse consequences to DLL4 inhibition are observed during clinical testing. Ongoing clinical studies will determine if this approach in blocking DLL4 can be developed into an effective therapeutic. As with all cancer therapeutics, identifying the right disease setting, dosing regimen, and combination strategy will be critical for successful drug development. It has been nearly 25 years since Notch was implicated in cancer by the discovery of Notch4/int-3 as a mammary proto-oncogene following activation by mouse mammary tumor virus (MMTV) integration [43,44]. With our increasing understanding of the function of this pathway in both normal cell fate decisions and in cancer, anti-DLL4 may become the prototype member of a new class of therapeutic agents.

## Competing interests

AG and TH are employees of OncoMed Pharmaceuticals which funds research described in this manuscript.

## Authors' contributions

AG and TH wrote the manuscript. All authors read and approved the final manuscript.

## References

[B1] Clevers H The cancer stem cell: premises, promises and challenges Nat Med 2011 17 313 319 21386835 10.1038/nm.2304

[B2] O'Brien CA Kreso A Jamieson CH Cancer stem cells and self-renewal Clin Cancer Res 2010 16 3113 3120 10.1158/1078-0432.CCR-09-2824 20530701

[B3] Clarke MF Self-renewal and solid-tumor stem cells Biol Blood Marrow Transplant 2005 11 14 16 15682169 10.1016/j.bbmt.2004.11.011

[B4] Barker N Ridgway RA van Es JH van de Wetering M Begthel H van den Born M Danenberg E Clarke AR Sansom OJ Clevers H Crypt stem cells as the cells-of-origin of intestinal cancer Nature 2009 457 608 611 10.1038/nature07602 19092804

[B5] Kume T Novel insights into the differential functions of Notch ligands in vascular formation J Angiogenes Res 2009 1 8 10.1186/2040-2384-1-8 20016694 PMC2794854

[B6] Gridley T Notch signaling in the vasculature Curr Top Dev Biol 2010 92 277 309 20816399 10.1016/S0070-2153(10)92009-7 PMC3659771

[B7] Roca C Adams RH Regulation of vascular morphogenesis by Notch signaling Genes Dev 2007 21 2511 2524 10.1101/gad.1589207 17938237

[B8] Hofmann JJ Iruela-Arispe ML Notch signaling in blood vessels: who is talking to whom about what? Circ Res 2007 100 1556 1568 10.1161/01.RES.0000266408.42939.e4 17556669

[B9] Liu H Zhang W Kennard S Caldwell RB Lilly B Notch3 is critical for proper angiogenesis and mural cell investment Circ Res 2010 107 860 870 10.1161/CIRCRESAHA.110.218271 20689064 PMC2948576

[B10] Liu H Kennard S Lilly B NOTCH3 expression is induced in mural cells through an autoregulatory loop that requires endothelial-expressed JAGGED1 Circ Res 2009 104 466 475 10.1161/CIRCRESAHA.108.184846 19150886 PMC2747310

[B11] Regan JN Majesky MW Building a vessel wall with notch signaling Circ Res 2009 104 419 421 10.1161/CIRCRESAHA.109.194233 19246684 PMC2727662

[B12] Dufraine J Funahashi Y Kitajewski J Notch signaling regulates tumor angiogenesis by diverse mechanisms Oncogene 2008 27 5132 5137 10.1038/onc.2008.227 18758482 PMC3893692

[B13] Real C Remedio L Caiado F Igreja C Borges C Trindade A Pinto-do OP Yagita H Duarte A Dias S Bone marrow-derived endothelial progenitors expressing delta-like 4 (dll4) regulate tumor angiogenesis PLoS One 2011 6 e18323 10.1371/journal.pone.0018323 21483741 PMC3070718

[B14] Milano J McKay J Dagenais C Foster-Brown L Pognan F Gadient R Jacobs RT Zacco A Greenberg B Ciaccio PJ Modulation of notch processing by gamma-secretase inhibitors causes intestinal goblet cell metaplasia and induction of genes known to specify gut secretory lineage differentiation Toxicol Sci 2004 82 341 358 10.1093/toxsci/kfh254 15319485

[B15] Imbimbo BP Therapeutic potential of gamma-secretase inhibitors and modulators Curr Top Med Chem 2008 8 54 61 10.2174/156802608783334015 18220933

[B16] Beel AJ Sanders CR Substrate specificity of gamma-secretase and other intramembrane proteases Cell Mol Life Sci 2008 65 1311 1334 10.1007/s00018-008-7462-2 18239854 PMC2569971

[B17] Hemming ML Elias JE Gygi SP Selkoe DJ Proteomic profiling of gamma-secretase substrates and mapping of substrate requirements PLoS Biol 2008 6 e257 10.1371/journal.pbio.0060257 18942891 PMC2570425

[B18] Bray SJ Notch signalling: a simple pathway becomes complex Nat Rev Mol Cell Biol 2006 7 678 689 10.1038/nrm2009 16921404

[B19] Benedito R Duarte A Expression of Dll4 during mouse embryogenesis suggests multiple developmental roles Gene Expr Patterns 2005 5 750 755 10.1016/j.modgep.2005.04.004 15923152

[B20] Duarte A Hirashima M Benedito R Trindade A Diniz P Bekman E Costa L Henrique D Rossant J Dosage-sensitive requirement for mouse Dll4 in artery development Genes Dev 2004 18 2474 2478 10.1101/gad.1239004 15466159 PMC529534

[B21] Gale NW Dominguez MG Noguera I Pan L Hughes V Valenzuela DM Murphy AJ Adams NC Lin HC Holash J Haploinsufficiency of delta-like 4 ligand results in embryonic lethality due to major defects in arterial and vascular development Proc Natl Acad Sci USA 2004 101 15949 15954 10.1073/pnas.0407290101 15520367 PMC524697

[B22] Krebs LT Shutter JR Tanigaki K Honjo T Stark KL Gridley T Haploinsufficient lethality and formation of arteriovenous malformations in Notch pathway mutants Genes Dev 2004 18 2469 2473 10.1101/gad.1239204 15466160 PMC529533

[B23] Koch U Fiorini E Benedito R Besseyrias V Schuster-Gossler K Pierres M Manley NR Duarte A Macdonald HR Radtke F Delta-like 4 is the essential, nonredundant ligand for Notch1 during thymic T cell lineage commitment J Exp Med 2008 205 2515 2523 10.1084/jem.20080829 18824585 PMC2571927

[B24] Hozumi K Mailhos C Negishi N Hirano K Yahata T Ando K Zuklys S Hollander GA Shima DT Habu S Delta-like 4 is indispensable in thymic environment specific for T cell development J Exp Med 2008 205 2507 2513 10.1084/jem.20080134 18824583 PMC2571926

[B25] Ito T Schaller M Hogaboam CM Standiford TJ Sandor M Lukacs NW Chensue SW Kunkel SL TLR9 regulates the mycobacteria-elicited pulmonary granulomatous immune response in mice through DC-derived Notch ligand delta-like 4 J Clin Invest 2009 119 33 46 19075396 10.1172/JCI35647 PMC2613456

[B26] Kassner N Krueger M Yagita H Dzionek A Hutloff A Kroczek R Scheffold A Rutz S Cutting edge: Plasmacytoid dendritic cells induce IL-10 production in T cells via the Delta-like-4/Notch axis J Immunol 2010 184 550 554 10.4049/jimmunol.0903152 20008296

[B27] Reynolds ND Lukacs NW Long N Karpus WJ Delta-Like Ligand 4 Regulates CNS T Cell Accumulation during Experimental Autoimmune Encephalomyelitis J Immunol 2011 10.4049/jimmunol.1100160 PMC3159801 21788444

[B28] Pellegrinet L Rodilla V Liu Z Chen S Koch U Espinosa L Kaestner KH Kopan R Lewis J Radtke F Dll1- and dll4-mediated notch signaling are required for homeostasis of intestinal stem cells Gastroenterology 2011 140 1230 1240 e1237 10.1053/j.gastro.2011.01.005 21238454 PMC3066401

[B29] Ridgway J Zhang G Wu Y Stawicki S Liang WC Chanthery Y Kowalski J Watts RJ Callahan C Kasman I Inhibition of Dll4 signalling inhibits tumour growth by deregulating angiogenesis Nature 2006 444 1083 1087 10.1038/nature05313 17183323

[B30] Noguera-Troise I Daly C Papadopoulos NJ Coetzee S Boland P Gale NW Lin HC Yancopoulos GD Thurston G Blockade of Dll4 inhibits tumour growth by promoting non-productive angiogenesis Nature 2006 444 1032 1037 10.1038/nature05355 17183313

[B31] Hoey T Yen WC Axelrod F Basi J Donigian L Dylla S Fitch-Bruhns M Lazetic S Park IK Sato A DLL4 blockade inhibits tumor growth and reduces tumor-initiating cell frequency Cell Stem Cell 2009 5 168 177 10.1016/j.stem.2009.05.019 19664991

[B32] Crawford Y Ferrara N VEGF inhibition: insights from preclinical and clinical studies Cell Tissue Res 2009 335 261 269 10.1007/s00441-008-0675-8 18766380

[B33] Sharma PS Sharma R Tyagi T VEGF/VEGFR Pathway Inhibitors as Anti-Angiogenic Agents: Present and Future Curr Cancer Drug Targets 2011 10.2174/156800911795655985 21486218

[B34] Hellstrom M Phng LK Hofmann JJ Wallgard E Coultas L Lindblom P Alva J Nilsson AK Karlsson L Gaiano N Dll4 signalling through Notch1 regulates formation of tip cells during angiogenesis Nature 2007 445 776 780 10.1038/nature05571 17259973

[B35] Funahashi Y Shawber CJ Sharma A Kanamaru E Choi YK Kitajewski J Notch modulates VEGF action in endothelial cells by inducing Matrix Metalloprotease activity Vasc Cell 2011 3 2 10.1186/2045-824X-3-2 21349159 PMC3039832

[B36] Mullendore ME Koorstra JB Li YM Offerhaus GJ Fan X Henderson CM Matsui W Eberhart CG Maitra A Feldmann G Ligand-dependent Notch signaling is involved in tumor initiation and tumor maintenance in pancreatic cancer Clin Cancer Res 2009 15 2291 2301 10.1158/1078-0432.CCR-08-2004 19258443 PMC2711441

[B37] Indraccolo S Minuzzo S Masiero M Pusceddu I Persano L Moserle L Reboldi A Favaro E Mecarozzi M Di Mario G Cross-talk between tumor and endothelial cells involving the Notch3-Dll4 interaction marks escape from tumor dormancy Cancer Res 2009 69 1314 1323 10.1158/0008-5472.CAN-08-2791 19208840

[B38] Martinez JC Muller MM Turley H Steers G Choteau L Li JL Sainson R Harris AL Pezzella F Gatter KC Nuclear and membrane expression of the angiogenesis regulator delta-like ligand 4 (DLL4) in normal and malignant human tissues Histopathology 2009 54 598 606 10.1111/j.1365-2559.2009.03279.x 19413639

[B39] Sato T van Es JH Snippert HJ Stange DE Vries RG van den Born M Barker N Shroyer NF van de Wetering M Clevers H Paneth cells constitute the niche for Lgr5 stem cells in intestinal crypts Nature 2011 469 415 418 10.1038/nature09637 21113151 PMC3547360

[B40] Fischer M Yen WC Kapoun AM Wang M O'Young G Lewicki J Gurney A Hoey T Anti-DLL4 inhibits growth and reduces tumor-initiating cell frequency in colorectal tumors with oncogenic KRAS mutations Cancer Res 2011 71 1520 1525 10.1158/0008-5472.CAN-10-2817 21193546

[B41] Yan M Callahan CA Beyer JC Allamneni KP Zhang G Ridgway JB Niessen K Plowman GD Chronic DLL4 blockade induces vascular neoplasms Nature 2010 463 E6 7 10.1038/nature08751 20147986

[B42] Djokovic D Trindade A Gigante J Badenes M Silva L Liu R Li X Gong M Krasnoperov V Gill PS Duarte A Combination of Dll4/Notch and Ephrin-B2/EphB4 targeted therapy is highly effective in disrupting tumor angiogenesis BMC Cancer 2010 10 641 10.1186/1471-2407-10-641 21092311 PMC3001720

[B43] Jhappan C Gallahan D Stahle C Chu E Smith GH Merlino G Callahan R Expression of an activated Notch-related int-3 transgene interferes with cell differentiation and induces neoplastic transformation in mammary and salivary glands Genes Dev 1992 6 345 355 10.1101/gad.6.3.345 1372276

[B44] Gallahan D Kozak C Callahan R A new common integration region (int-3) for mouse mammary tumor virus on mouse chromosome 17 J Virol 1987 61 218 220 3023699 10.1128/jvi.61.1.218-220.1987 PMC255246

